# Supplementary material for: Causal Relationship between Aging and Anorexia Nervosa: A White-Matter-Microstructure-Mediated Mendelian Randomization Analysis
Source: Biomedicines. 2024 Aug 16;12(8):1874. doi: 10.3390/biomedicines12081874 (PMC11351342; doi:10.3390/biomedicines12081874)
Supplement: Supplementary file 1 [file biomedicines-12-01874-s001.zip › Supplementary Figure/Figure S8/Leaveoneout plot of MR analysis for TL to ubm-b-1971.pdf]

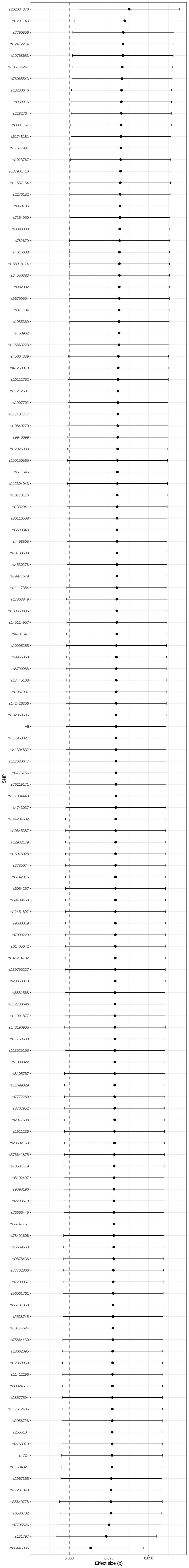

rs202034370
rs1291143
rs7790856
rs12412214
rs10768683
rs185174247
rs76065543
rs13230646
rs939916
rs2282764
rs3891167
rs61748181
rs17677991
rs1023767
rs137901416
rs11557154
rs2276182
rs869785
rs7164950
rs3093888
rs762679
rs4616688
rs188918174
rs34550383
rs932002
rs56799554
rs871134
rs1985369
rs450962
rs116863223
rs45604339
rs41269079
rs10112752
rs11212631
rs1907702
rs117407747
rs10840270
rs9940099
rs12925933
rs150150565
rs611646
rs112394943
rs10773176
rs1332941
rs80116508
rs6590343
rs4498805
rs73730598
rs4530278
rs79977579
rs11117354
rs17803849
rs139669835
rs145114957
rs4731541
rs10905255
rs9955360
rs6790988
rs17445108
rs1957937
rs142426306
rs182059586
A8
rs111950327
rs41304832
rs117630647
rs6776756
rs76219171
rs117034449
rs4743037
rs144204502
rs10845367
rs12932178
rs16978028
rs3785074
rs5742915
rs6054257
rs59409453
rs12451892
rs9600019
rs7099229
rs61405042
rs141214782
rs139795227
rs28363070
rs6881568
rs142730696
rs11991877
rs143190905
rs11769630
rs113525195
rs1003322
rs8105767
rs11699829
rs7772289
rs3767952
rs2977608
rs1611236
rs28502153
rs376641875
rs73581419
rs8102497
rs9398196
rs2293579
rs76666449
rs55747751
rs78491606
rs6669563
rs9878436
rs77732866
rs7209057
rs56061761
rs66731853
rs2538745
rs10774624
rs75664430
rs13062095
rs12369950
rs11412296
rs80324517
rs28577594
rs117512405
rs2056726
rs2555104
rs2763979
rs4724
rs11584821
rs2967355
rs77231040
rs35640778
rs6536702
rs7705526
rs131797
rs35446936
SNP
0.000
0.025
0.050
Effect size (b)
